# Low albumin level is more strongly associated with adverse outcomes and *Staphylococcus aureus* infection than hemoglobin A1C or smoking tobacco

**DOI:** 10.1002/jor.25282

**Published:** 2022-02-04

**Authors:** Michael P. Campbell, Makinzie D. Mott, John R. Owen, Julie E. Reznicek, Christopher A. Beck, Gowrishankar Muthukrishnan, Gregory J. Golladay, Stephen L. Kates

**Affiliations:** ^1^ Department of Orthopaedic Surgery Virginia Commonwealth University Richmond Virginia USA; ^2^ Department of Pathology University of Louisville Louisville Kentucky USA; ^3^ Department of Infectious Disease Virginia Commonwealth University Richmond Virginia USA; ^4^ Center for Musculoskeletal Research University of Rochester Medical Center Rochester New York USA; ^5^ Department of Biostatistics and Computational Biology University of Rochester Medical Center Rochester New York USA

**Keywords:** albumin, antibiotics, immunoassay, orthopaedic infections, osteomyelitis, serum anti‐Staphylococcal antibodies, *Staphylococcus aureus*

## Abstract

Postsurgical deep musculoskeletal infections are a major clinical problem in Orthopaedic Surgery. A serum‐based nomogram, which can objectively risk‐stratify patients, and aid surgeons in delineating infection risk associated with orthopedic surgical interventions, would be immensely helpful. Here, we constructed a multi‐parametric nomogram based on serum anti‐*Staphylococcus aureus* antibody responses, patient characteristics including demographics and standard clinical tests. This nomogram was formally tested in a prospective cohort study comparing 303 hospitalized patients with culture‐confirmed *S. aureus* infection compared with a cohort of 223 healthy screened preoperative patients. Serum anti‐*S. aureus* antibody responses, standard of care clinical tests, and patient demographic data were utilized to perform multivariate logistic regression analysis to quantify the presence of infection and adverse outcome using odds ratios (OR) and to assess predictive ability via area under the ROC curve (AUC). At enrollment, high anti‐*S. aureus* IgG titers were predictive of infection. Remarkably, low serum albumin was found to be significantly associated with infection (OR = 479.963, 95% CI 61.59 ‐ 3740.33, *p* < 0.0001) and this finding was surprisingly higher than BMI or HbA1c‐associations. Combining all risk factors in the nomogram yielded a diagnostic AUC of 0.949 for predicting *S. aureus* infection. Our results indicate that a serum‐based multi‐parametric nomogram can be useful in diagnosing *S. aureus* infections, and importantly, malnourishment is significantly associated with these infections.

## 1 INTRODUCTION

Deep musculoskeletal infections after orthopedic operations represent a major clinical problem and are gradually increasing each year with increasing surgical volume.^1^ The most commonly isolated pathogen in orthopedic infection is *Staphylococcus aureus*.^2^ Overall, Methicillin‐resistant *Staphylococcus aureus* infects 120,000 patients with bloodstream infections and contributes to 20,000 deaths annually.^3^ With an increasing number of joint arthroplasties performed in the United States, there has been a commensurate increase in peri‐prosthetic joint infection (PJI) which places a large economic burden on the American health care system, costing $566 million in 2009.^4^ *S. aureus* is the leading cause of PJI.^5^ With rising antibiotic resistance, a need exists for nonantibiotic, immune‐based approaches to treat drug‐resistant infections.^6^

Host factors play a key role in infection, as we have been unable to reduce infection rates for elective procedures below 1%–2%.^7^ Many patient‐specific factors seem to influence susceptibility to infection, including comorbid conditions^8^ such as obesity and diabetes, all of which can affect immune responses to *S. aureus* infection.^9^ According to the 2018 International Consensus Meeting on Musculoskeletal Infection, high BMI received unanimous agreement as a risk factor for surgical site infection and PJI.^10^ Albumin is a commonly used marker for nutritional status. Serum albumin level >3.5 g/dl is frequently used as an optimization parameter in the clinic before major elective surgery.^10^

We have previously identified multiple immunodominant *S. aureus* antigens, which provoke robust antibody responses in patients with musculoskeletal infections. Antibody titers against these *S. aureus* antigens can be measured by immunoassay of a patient's sera.^11^ To date, the only way to confirm the diagnosis of deep musculoskeletal infection with *S. aureus* is to obtain a fluid/tissue sample followed by microbial culture, which can be a lengthy process sometimes limited by culture‐negative results. There is no currently accepted diagnostic blood test to confirm infection with *S. aureus*. Such a diagnostic test would be invaluable to surgeons, as it would eliminate the need to perform invasive procedures for diagnosing deep infections. Additionally, the ability to risk‐stratify patients based upon laboratory values and patient characteristics could help surgeons identify patients at increased infection risk associated with orthopedic surgery.

We hypothesize that host factors, notably high BMI, and hemoglobin A1c, contribute to the risk of developing a *Staphylococcus aureus* infection. By measuring antibody levels in individuals infected with *S. aureus*, we can quantify the humoral immune response to *S. aureus* in individuals. We hope to create a combined nomogram to predict infection risk patients undergoing orthopedic procedures. The aims of our study are (1) to analyze and compare anti‐*S. aureus* humoral immune responses in patients with *S. aureus* infections to individuals without infection, by measuring their antibodies. (2) To produce a multiparametric nomogram based upon host factors and *anti‐S. aureus* antibodies that would give a risk‐adjustment for *S. aureus* infection in patients undergoing orthopedic procedures.

## 2 MATERIALS AND METHODS

### 2.1 Study design

This Level 2 prospective cohort study was approved by the University Institutional Review Board. Inclusion criteria included hospitalized patients ≥18 years old with culture‐confirmed *S. aureus* (wound culture, blood culture). For the control group, inclusion criteria included healthy, infection‐free patients ≥18 years old undergoing elective orthopedic surgery. We excluded prisoners, patients undergoing active cancer treatment, and patients with a history of solid organ transplant on immunosuppressive medications. Patient demographic data (age, sex, race, body mass index, smoking status) and laboratory data (hemoglobin A1C, albumin) were collected. For the patients with infection, infection location (bone, pulmonary, endocarditis, bacteremia of unknown origin), and resistance or sensitivity of the cultured *S. aureus* to methicillin was collected. All samples were collected before December 2019, before the onset of the COVID‐19 pandemic to avoid possible confounding effects of COVID‐19 on the immune system.

## 3 CLINICAL OUTCOMES

Outcomes were categorized as good, adverse, or unknown. A good outcome was defined as “infection control.” An adverse outcome was defined as death, persistent infection despite treatment, amputation, or joint fusion. Outcomes were unknown if there was no documented follow‐up at one year.

## 4 SEROLOGY

Serum anti‐*S. aureus* IgG antibodies were measured using a custom multiplex Luminex assay following the methodology previously reported.^12^, ^13^, ^14^, ^15^, ^16^, ^17^ Eight immunodominant *S. aureus* antigens were measured; three iron‐regulated surface determinant (Isd) proteins: (IsdA, IsdB, and IsdH), three secretory proteins: chemotaxis inhibitory protein from *S. aureus* (CHIPS), α‐hemolysin (Hla), and staphylococcal complement inhibitor (SCIN), and two cell wall autolysin (Atl) domains: amidase (Amd) and glucosaminidase (Gmd). Serum IgG titers are reported as median fluorescent intensity (MFI) units for each antigen at a constant serum dilution level.

As in previous studies, each biotinylated antigen was coupled at a density of 50 pmol/million beads to distinct bead regions of avidin‐coated magnetic beads (MagPlex‐Avidin, Luminex Corp.). Fifty microliters containing 1000 coupled beads were then mixed with 50 µl of 1:5000 diluted sera per well yielding 100 µl of 1:10,000 diluted sera with 1000 beads per well and were incubated for two hours while shaking on a microplate shaker at room temperature. Afterward, a wash step removed unbound sera, 100 µl of 1:500 diluted phycoerythrin‐conjugated goat anti‐human IgG reagent (Cat. #2040‐09, Southern Biotech) was added as the detection antibody and the plate was incubated for 1 h while shaking at room temperature. The plate was then washed to remove unbound detection antibodies and 130 µl of PBST‐BSA (phosphate‐buffered saline with 0.1% bovine serum albumin and 0.1% TWEEN) was added to each well. Finally, the beads were resuspended by 2 min of shaking and mixing by pipette before measurement of IgG bound to each antigen via Luminex (Luminex 200, xPONENT v3.1, Luminex Corp.).

## 5 STATISTICS

Patient characteristics were compared between the infected and uninfected patients, with further categorization based on body mass index, age, sex, smoking status, hemoglobin A1c, and albumin. Anti‐*S. aureus* IgG titers were categorized based on data quartiles as Low (below the first quartile), Intermediate (between the first and third quartiles), and High (above the third quartile). Infection rates and adverse outcome rates were compared using Fisher's exact test for High versus Low and using Cochran–Armitage exact trend tests across Low, Intermediate, and High. The odds of infection and adverse outcome were modeled using univariate and multivariate logistic regression models with risk characterized using odds ratios (OR) with 95% confidence intervals and p‐values. Models treating IgG titers as continuous predictors utilized base‐10 log‐transformations to reduce skewness and improve model fit, resulting in ORs per 10‐fold increase in antibody titers values. For simplicity and clinical relevance, multivariate models used predictors that were dichotomized based on the category of greatest risk (e.g., High vs. not High). Clinical and serologic risk factors were assessed for their predictive ability in discriminating infection and adverse outcome status using receiver operating characteristic (ROC) curve analysis, with overall prediction accuracy summarized by the area under the ROC curve (AUC). All analyses were performed using SAS version 9.4 and R version 3.5.1. A *p*‐value less than 0.05 was considered significant.

## 6 RESULTS

### 6.1 Patient demographics and infection characterization

In total, 223 control specimens from healthy individuals and 303 specimens from *S. aureus*‐infected patients were collected. Demographic and laboratory data, infection type, sensitivity to methicillin, and clinical outcomes for infection patients are shown in Table 1.

**Table 1:** Demographic data for infection and control sample counts (percentage) along with infection status, infection type, methicillin sensitivity, and outcomes of infected patients

Gender		Age		Smoker		BMI		HbA1c	
Male	266 (50.6%)	18 to <30	34 (6.5%)	Yes	142 (27.0%)	<30	290 (55.2%)	<7	272 (51.7%)
Female	260 (49.4%)	30–70	428 (81.3%)	No	378 (71.9%)	30 to <40	178 (33.8%)	≥7	80 (15.2%)
Total	526 (100%)	>70	64 (12.2%)	Unknown	6 (1.1%)	≥40	58 (11.0%)	Unknown	174 (33.1%)
		Total	526 (100%)	Total	526 (100%)	Total	526 (100%)	Total	526 (100%)

Albumin		Infection status		MRSA vs. MSSA		Infection type		Outcome	
<2.5	67 (12.7%)	Control	223 (42.4%)	MSSA	172 (56.8%)	Bacteremia	120 (39.6%)	Good	114 (37.6%)
≥2.5 to <3.5	134 (25.5%)	Infected	303 (57.6%)	MRSA	131 (43.2%)	Bone/Joint	133 (43.9%)	Adverse	160 (52.8%)
≥3.5	210 (39.9%)	Total	526 (100%)	Total	303 (100%)	Endocarditis	37 (12.2%)	Unknown	29 (9.6%)
Unknown	115 (21.9%)					Lung	13 (4.3%)	Total	303 (100%)
Total	526 (100%)					Total	303 (100%)		

### 6.2 Humoral response and patient factors associated with infection

**Figure 1:**
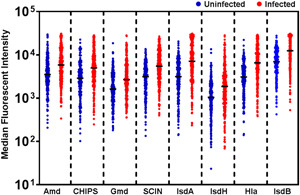
Median fluorescent intensity was higher for *Staphylococcus* aureus‐infected patients than uninfected patients. Sera from uninfected (*n* = 223) and *S*. aureus‐infected (*n* = 303) patients were analyzed via Luminex custom assay for IgG antibody median fluorescent intensity levels against eight antigens: Amd, CHIPS, Gmd, SCIN, IsdA, IsdH, Hla, and IsdB. Horizontal black bars represent median values for each antigen [Color figure can be viewed at wileyonlinelibrary.com]

The sera for all 526 patients were tested for antibody responses against these aforementioned *S*. *aureus* antigens in our custom Luminex immunoassay. Interestingly, we observed that high levels of serum IgG titers were associated with the presence of infection (Table 2; Figure 1). When comparing clinical factors, low versus high albumin was found to have an OR 200.765 (95% CI 44.62 to infinite, *p* < 0.0001) (Table 3). A multivariate logistic regression analysis was performed for infection (Table 4). Remarkably, low albumin levels in patients were highly associated with *S. aureus* infection with an OR of 539.205 (95% CI 71.69–4055.44; *p* < 0.0001). When combining clinical and serologic patient factors, the area under the curve was 0.949, which suggests a high predictive value for *S. aureus* infection.

**Table 2:** Logistic regression analysis for each Antigen showing increased risk of Infection and Adverse Outcomes per 10‐fold increase in antibody levels

	Infection risk, OR (95% CI)	Adverse outcomes, OR (95% CI)
Amd	**4.160** **** (2.57, 6.72)	**3.019** *** (1.57, 5.82)
CHIPS	**4.592** **** (2.91, 7.25)	**2.468** ** (1.35, 4.50)
Gmd	**4.618** **** (2.86, 7.45)	**2.967** *** (1.57, 5.60)
SCIN	**7.131** **** (4.28, 11.87)	**3.448** **** (1.76, 6.77)
IsdA	**6.391** **** (4.12, 9.92)	**2.222** ** (1.28, 3.86)
IsdH	**2.985** **** (2.02, 4.40)	**2.475** *** (1.47, 4.15)
Hla	**6.625** **** (4.12, 10.65)	**3.873** **** (2.11, 7.12)
IsdB	**7.837** **** (4.46, 13.78)	**3.962** **** (1.91, 8.24)

** *p* < 0.01.

*** *p* < 0.001.

**** *p* < 0.0001.

**Table 3:** Risk of infection risk and adverse outcomes by other potential risk factors

	Infection risk	Adverse outcomes
	OR (95% CI)	OR (95% CI)
**BMI**		
High vs. Normal	0.975 (0.54, 1.77)	2.189 (0.78, 6.15)
Low vs. Normal	**2.192** **** (1.50, 3.21)	1.034 (0.60, 1.77)
High vs. Low	**0.445** ** (0.25, 0.79)	2.118 (0.80, 5.63)
**Age**		
>70 vs. <30	**0.371** * (0.15, 0.94)	1.75 (0.58, 5.32)
30–70 vs. <30	**0.400** * (0.18, 0.90)	**2.345** * (1.01, 5.45)
>70 vs. 30–70	0.928 (0.55, 1.57)	0.746 (0.33, 1.69)
**Sex**		
Male vs. female	**1.983** **** (1.40, 2.82)	1.172 (0.72, 1.91)
**Smoking**		
Smoker vs nonsmoker	**2.883** **** (1.88, 4.42)	1.113 (0.67, 1.84)
**HbA1C**		
High vs. Normal	**1.771** * (1.05, 2.98)	1.386 (0.68, 2.84)
**Albumin**		
High vs. Intermediate	**0.014** **** (0.01, 0.04)	**0.501** * (0.27, 0.93)
Low vs. Intermediate	2.689 (0.45, Inf)	**2.482** * (1.21, 5.10)
Low vs. High	**200.765** **** (44.62, Inf)	**4.954** **** (2.25, 10.91)

* *p* < 0.05.

** *p* < 0.01.

**** *p* < 0.0001.

**Table 4:** Risk of infection and adverse outcomes considering all eight *Staphylococcus aureus* antigens without risk factors, all six risk factors alone, and all antigens and risk factors combined

	Risk of infection			Risk of adverse outcomes		
	Antigens only (*N* = 526)	Risk factors onl y (*N* = 305)	Antigens and risk facto rs (*N* = 305)	Antigens only (*N* = 274)	Risk factors o nly (*N* = 159)	Antigens and risk fact ors (*N* = 159)
AUC	0.751	0.936	0.949	0.669	0.666	0.811
Parameters	OR (95% CI)	OR (95% CI)	OR (95% CI)	OR (95% CI)	OR (95% CI)	OR (95% CI)
Amd not high	1.185 (0.56, 2.50)		0.607 (0.16, 2.37)	0.506 (0.22, 1.15)		0.391 (0.12, 1.26)
CHIPS high	1.332 (0.74, 2.39)		1.245 (0.34, 4.61)	0.905 (0.43, 1.92)		2.806 (0.79, 9.93)
Gmd not high	1.613 (0.74, 3.51)		2.25 (0.47, 10.8)	1.331 (0.54, 3.25)		1.223 (0.37, 4.07)
SCIN not high	1.032 (0.50, 2.13)		0.572 (0.15, 2.24)	0.980 (0.42, 2.31)		1.370 (0.32, 5.96)
IsdA high	**3.685** ** (1.72, 7.90)		0.861 (0.18, 4.21)	0.845 (0.36, 2.01)		0.426 (0.09, 2.04)
IsdH high	1.292 (0.70, 2.37)		0.941 (0.27, 3.27)	0.700 (0.30, 1.65)		1.115 (0.31, 4.03)
Hla high	**6.667** **** (3.14, 14.16)		3.18 (0.71, 14.19)	**3.968** ** (1.71, 9.21)		**5.946** * (1.40, 25.25)
IsdB high	**2.769** ** (1.35, 5.67)		3.43 (0.96, 12.22)	2.152 (0.87, 5.35)		2.831 (0.66, 12.23)
Low BMI		1.378 (0.61, 3.09)	1.439 (0.60, 3.47)		0.336 (0.09, 1.23)	0.292 (0.07, 1.23)
Age,<30		1.716 (0.23, 12.98)	3.008 (0.38, 24.04)		0.417 (0.10, 1.66)	0.489 (0.10, 2.32)
Male		1.259 (0.56, 2.83)	1.012 (0.41, 2.49)		**2.117** * (1.03, 4.34)	1.942 (0.87, 4.32)
Smoker		**2.921** * (1.04, 8.19)	2.924 (0.93, 9.24)		**2.371** * (1.00, 5.60)	1.951 (0.78, 4.89)
Diabetes		2.002 (0.80, 5.00)	2.138 (0.76, 6.03)		1.267 (0.58, 2.78)	1.458 (0.60, 3.55)
Albumin not high		**539.205** **** (71.69, 4055.44)	**479.963** **** (61.59, 3740.33)		**2.936** * (1.22, 7.04)	**2.678** * (1.00, 7.16)

* *p* < 0.05.

** *p* < 0.01.

**** *p* < 0.0001.

### 6.3 Humoral response and patient factors associated with adverse outcome

High levels of all antigens were also predictive of adverse outcome (Table 2). Low albumin compared to intermediate and high albumin increased risk of adverse outcome (Table 3). A multivariate logistic regression analysis was performed for adverse outcome (Table 4). When considering antigens alone and clinical risk factors alone, high H1a and low albumin predicted adverse outcome with odds ratios of 3.968 (95% CI 1.71–9.21; *p* < 0.01) and 2.936 (95% CI 1.22–7.01; *p* < 0.05) respectively. With antigens and risk factors combined, Hla still predicted adverse outcomes with an odds ratio of 5.946 (95% CI 1.40–25.25; *p* < 0.05) and low albumin indicated adverse outcomes with an odds ratio of 2.678 (95% CI 1.00 to 7.16; *p* = 0.050).

### 6.4 Additive risk factors associated with increased infection and adverse outcomes

With an increasing number of risk factors, the odds ratio of infection increased (Table 5). The same is true of the adverse outcomes (Table 6).

**Table 5:** Risk of infection increases as additional antigens and risk factors are incrementally added

Number of parameters in combination	Risk factor	OR (95% CI)	Antigen	OR (95% CI)	Risk factor + antigen	OR (95% CI)
1	Low Albumin	**539.20** **** (71.69, 4055.44)	High Hla	**6.67** **** (3.14, 14.16)	Low Albumin	**479.96** **** (61.59, 3740.33)
2	plus Smoker	**1574.93** **** (157.23, 15775.20)	plus High IsdA	**24.57** **** (8.20, 73.62)	plus High IsdB	**1646.34** **** (134.19, 20197.85)
3	plus Diabetes	**3152.45** **** (248.93, 39922.77)	plus High IsdB	**68.03** **** (20.52, 225.53)	plus High Hla	**5235.73** **** (297.73, 92071.77)
4	plus Age <30	**5409.66** **** (249.80, 117152.85)	plus Not High Gmd	**109.70** **** (23.93, 502.80)	plus Age <30	**15746.94** **** (390.90, 634344.86)
5	plus Low BMI	**7452.13** **** (325.21, 170765.34)	plus High CHIPS	**146.16** **** (30.04, 711.06)	plus Smoker	**46041.93** **** (1025.68, 2066791.36)

*Note*: The top five parameters which were most impactful are shown when considering only risk factors, only antigens, or a combination of both. The combination of risk factors and antigens has the greatest impact.

**** *p* < 0.0001.

**Table 6:** Risk of adverse outcomes increases as additional antigens and risk factors are incrementally added

Number of parameters in combination	Risk factor	OR (95% CI)	Antigen	OR (95% CI)	Risk factor + antigen	OR (95% CI)
1	High BMI	2.98 (0.82, 10.86)	High Hla	**3.97** ** (1.71, 9.21)	High Hla	**5.95** * (1.40, 25.25)
2	plus Low Albumin	**8.74** * (1.64, 46.51)	plus High IsdB	**8.54** ** (2.36, 30.92)	plus High BMI	**20.38** ** (2.48, 167.65)
3	plus Age ≥30	**20.94** ** (2.33, 187.91)	plus High Amd	**16.87** *** (3.73, 76.30)	plus High IsdB	**57.71** ** (4.54, 733.47)
4	plus Smoker	**49.65** ** (3.73, 660.48)	plus Not High IsdH	**24.12** ** (3.62, 160.77)	plus High CHIPS	**161.92** *** (10.30, 2545.49)
5	plus Male	**105.10** ** (6.52, 1695.11)	plus Not High Gmd	**32.09** ** (3.34, 308.45)	plus Low Albumin	**433.55** *** (20.23, 9289.73)

*Note*: The top five parameter combinations which were most impactful are shown when considering only risk factors, only antigens, or a combination of both. The combination of risk factors and antigens has the greatest impact.

* *p* < 0.05.

** *p* < 0.01.

*** *p* < 0.001.

## 7 DISCUSSION

The primary aim of this study was to analyze and compare the immune response of patients infected with *S. aureus* and those individuals not infected, by measuring their anti‐staphylococcal antibodies. We hypothesized that host factors, notably high BMI and Hemoglobin A1c, contribute to *S. aureus* infections. We were able to quantify the immune response to *S. aureus* infections, demonstrating that high levels of all antigens tested increased odds of infection. Additionally, these data show the tremendous increase in infection risk with low albumin. This suggests the significant role that malnourishment plays in susceptibility to *S. aureus* infection. High hemoglobin A1c did contribute, but high BMI did not. By combing all clinical and serologic host factors, we were able to achieve an AUC of 0.949 for predicting *S. aureus* infection.

High hemoglobin A1c does increase the risk of *S. aureus* infection, but high BMI was not associated with increased infection risk in this sample of patients. Surprisingly, low albumin had a more significant impact on infection risk with an OR of 200 when comparing low versus high albumin. Thus, we disproved our hypothesis.

The secondary aim of this study was to produce a nomogram based upon host factors that would give a risk adjustment for *S. aureus* infection for patients undergoing orthopedic procedures. By combining all host factors, we were able to achieve a high AUC of 0.949 for identifying *S. aureus* infections.

Humoral response to *S. aureus* infection has been previously studied. Our previous studies on serum samples obtained from patients with confirmed *S. aureus* bone infections (AO Trauma CPP Bone Infection Registry^13^) revealed that anti‐IsdB IgG titers are predictive of infection.^11^ Interestingly, higher levels of IgG against autolysin enzymes (Amd, Gmd) and secretory immune evasion proteins (Hla, SCIN, and CHIPS) were also significant predictors of infection control and positive control in these patients.^11^ Though infection risk was not directly calculated in the aforementioned study, we observed that the adverse outcome risk is more significant in this study. Jacobsson et al.^18^ evaluated a prospective cohort of patients infected with *S. aureus* compared to a control group. The antigens they evaluated included ribitol teichoic acid, alpha‐toxin, enterotoxin A, toxic shock syndrome toxin 1, scalded skin syndrome toxin, lipase, clumping factor A, and extracellular fibrinogen binding. They noted that patients with a fatal outcome demonstrated significantly lower antibody levels against teichoic acid, lipase, enterotoxin A, and scalded skin syndrome toxin. They did not evaluate potential comorbid risk factors and their associations with humoral response. It is difficult to compare our study to Jacobsson et al. because different antigens were evaluated.^18^ In our study, we found that low albumin was associated with *S. aureus* infection and adverse outcome.

Risk factors for *S. aureus* infection have been previously evaluated through epidemiologic analysis. The review by van Hal et al.^19^ notes age as the strongest predictor of mortality in *S. aureus* bacteremia. Analysis of age as a risk factor in our study population revealed contradictory results. Only low albumin was most strongly associated with infection risk and adverse outcome. Of note, our infection population is a heterogeneous group, whereas the review by van Hal et al. only reviewed patients with bacteremia.^19^

There are several strengths of our study. It is prospective with large sample size and uses both clinical demographic data with quantifiable humoral immune response measurements to analyze *S. aureus* infection risk. Our data were collected before the COVID‐19 pandemic. This is a tremendous strength, as we do not know how COVID infection may alter the immune system, which could act as a confounding variable. However, our study has limitations. The infection group is a heterogeneous population of hospitalized patients, not just orthopedic‐related infections. This heterogeneous population may also be considered a strength. Additionally, there was some missing data ‐not all laboratory data (hemoglobin A1c, albumin) or outcome data were available for every patient.

## 8 CONCLUSIONS

Of the host risk factors evaluated, low albumin was most strongly associated with *S. aureus* infection and adverse outcome—more so than high BMI or hemoglobin A1c. By combining all host risk factors, we were able to achieve an AUC of ∼0.95 for predicting *S. aureus* infection.

## AUTHOR CONTRIBUTIONS

Michael P. Campbell: project design, sample collection, data analysis, interpretation, drafting manuscript. Makinzie D. Mott; project design, sample collection, sample analysis. John R. Owen: project design, sample collection, sample analysis, statistical analysis. Julie E. Reznicek: project design, sample identification, and collection. Christopher A. Beck: complex data analysis and interpretation, drafting manuscript. Gowrishankar Muthukrishnan: data analysis and interpretation, drafting manuscript. Gregory G. Golladay: project design, sample collection. Stephen L. Kates: project design, funding, data analysis and interpretation, drafting manuscript.
